# Outcomes of direct superior approach and posterolateral approach for hemiarthroplasty in the treatment of elderly patients with displaced femoral neck fractures: A comparative study

**DOI:** 10.3389/fsurg.2023.1087338

**Published:** 2023-03-14

**Authors:** Wei Hu, Wen-Bo Xu, Hao Li, Wen-Hua Jiang, Yin-Chu Shao, Ji-Chun Shan, Di Yang, De-En Wan, Feng Shuang

**Affiliations:** Department of Orthopaedics, The 908th Hospital of the Joint Logistic Support Force of the Chinese People's Liberation Army, Nanchang, China

**Keywords:** displaced femoral neck fractures, hemiarthroplasty, direct superior approach, posterolateral approach, elderly patients

## Abstract

Hemiarthroplasty is a surgical choice for super-aged patients with a high surgical risk and a sedentary lifestyle. The direct superior approach (DSA), a minimally invasive modification of the posterior approach, is rarely studied in hemiarthroplasty. The aim of the present study was to compare the clinical outcomes in elderly patients with displaced femoral neck fractures undergoing hemiarthroplasty *via* DSA with the conventional posterolateral approach (PLA). A total of 48 elderly patients with displaced femoral neck fractures who underwent hemiarthroplasty between February 2020 and March 2021 were retrospectively included in the study. Of them, 24 patients (mean age 84.54 ± 2.11 years) were treated with hemiarthroplasty *via* DSA (DSA group), while the other 24 patients (mean age 84.92 ± 2.15 years) were treated with hemiarthroplasty *via* PLA (PLA group). Clinical outcomes, perioperative data, and complications were recorded. There were no obvious differences in the baseline characteristics between the DSA and PLA groups, including age, gender, body mass index, Garden type, American Society of Anesthesiologists score, and hematocrit. Perioperative data showed that the length of the incision in the DSA group was smaller than that in the PLA group (*p* < 0.001). However, the duration of the operation and blood loss in the DSA group were longer and higher than those in the PLA group, respectively (*p* < 0.001). In addition, the DSA group had a shorter hospitalization time than the PLA group (*p* < 0.001). The visual analog scale score and Harris score 1 month postoperatively in the DSA group were better than those in the PLA group (*p* < 0.001). Moreover, there were no significant differences between the two groups in Harris score (for assessment dysfunction) 6 months postoperatively (*p* > 0.05). DSA is less invasive and has better clinical outcomes, which can allow an early return to daily living activities in elderly patients with displaced femoral neck fractures undergoing hemiarthroplasty.

## Introduction

With the aging of the global population, the prevalence of femoral neck fractures increases proportionately (1). Femoral neck fractures, the most common hip-related fractures, account for almost 50% of hip fractures (2). Femoral neck fractures in the elderly population are associated with a higher mortality rate due to multiple co-morbidities (3). Thus, seeking adequate interventions and effective therapy is essential to enhance the outcomes of femoral neck fractures in the elderly population.

At present, the commonly used treatment options for femoral neck fractures include conservative treatment, internal fixation, and replacement surgery (4, 5). In general, internal fixation is effective for patients with non-displaced fractures (Garden type I or II fractures) (6), whereas hemiarthroplasty and total hip arthroplasty are common approaches for patients with displaced fractures (Garden type III or IV fractures) (5). However, a previous study has found that revision surgery occurs frequently after treating internal fixation (7). In addition, accumulating evidence has demonstrated that hemiarthroplasty and total hip arthroplasty are better than internal fixation in terms of recovery of hip function, quality of life, and reoperation rates (8–10). The choice between hemiarthroplasty and total hip arthroplasty depends mainly on the patient’s physical active needs, age, and physical condition (11). Moreover, Lu et al. (12) have clarified that hemiarthroplasty is a good choice for the treatment of super-aged patients with a high surgical risk of and sedentary lifestyle.

However, choosing surgical interventions is still a challenge for orthopedists because of the high morbidity rate and elevated costs. It is well known that surgical approaches for hip replacements can influence clinical outcomes (13). The direct superior approach (DSA), a minimally invasive modification of the posterior approach, has been proven to have potential benefits in terms of dislocation rate and postoperative recovery (14). In addition, the conventional posterolateral approach (PLA) is possibly the most common approach for hip replacements, which can provide optimal visualization (15, 16). When comparing DSA and PLA, a previous study stated that DSA is superior to PLA regarding the recovery of total hip arthroplasty (17). However, there are limited studies comparing the clinical efficacy of DSA versus PLA for hemiarthroplasty in patients with displaced femoral neck fractures.

The aim of the present study was to evaluate the clinical outcomes of DSA in elderly patients with displaced femoral neck fractures undergoing hemiarthroplasty in comparison with PLA. These findings may provide guidance for the management of displaced femoral neck fractures.

## Material and methods

### Patients

Patients with femoral neck fractures undergoing hemiarthroplasty at the 908th Hospital of the Joint Logistic Support Force of the People’s Liberation Army between February 2020 and March 2021 were included retrospectively.

The inclusion criteria were as follows: (1) patients with Garden type III or IV fractures; (2) patients aged older than 75 years; (3) patients who underwent hemiarthroplasty for the first time; (4) patients who underwent unilateral surgery; and (5) patients who were able to cooperate with the examination and follow-up. The exclusion criteria were as follows: (1) patients with severe underlying diseases who could not tolerate the anesthesia and surgical risks; (2) patients with a history of hip surgery; (3) patients with severe osteoporosis; and (4) patients who did not cooperate with the follow-up.

According to the surgical approach, patients who underwent the hemiarthroplasty *via* DSA were defined as the DSA group, while patients who underwent hemiarthroplasty *via* PLA were defined as the PLA group. The study was approved by the ethical committee of the 908th Hospital of the Joint Logistic Support Force of the People's Liberation Army (No. 2020LL007), and methods were performed in accordance with Helsinki guidelines. Since the data were retrospectively collected, informed consent could not be obtained.

### Surgical procedure

In the DSA group, the patient was placed in a semi-recumbent position with the affected hip facing upward. After routine disinfection, a protective film of incision was attached. A 5-cm incision was made directly above the affected hip joint, which was located slightly behind the apex of greater trochanter, 1 cm below the apex and 4 cm above the apex. The skin, subcutaneous tissue, and gluteus musculature were incised, and the gluteus maximus fibers were then separated anterogradely. Next, the gluteus medius muscle was separated by the automatic retractor and fat tissue could be seen. A periosteal stripper was performed to expose the gluteus minimus behind the gluteus medius and the piriformis below the gluteus medius. After exposing the joint capsule above the hip joint, the joint capsule was cut open and the sutures were marked for suturing to protect the short external rotator muscle group of the joint capsule. If it was difficult to expose, flexion, adduction, and internal rotation of the hip at the insertion point of the piriformis muscle was cut. Subsequently, the femoral neck at the base of femoral neck was severed, leaving about 1.0 cm of the femoral talus. The femoral head was removed and the maximum diameter was measured. On the osteotomy surface of the femoral neck, a grooving device with an anteversion angle was utilized to open the femur, and the medullary cavity was grinded and reamed from small to large. After installing the appropriately sized femoral prosthesis and femoral head, the hip joint was reset. The extension, rotation, adduction, and abduction movements, the stability of the prosthesis, and the function of the hip joint were checked. When there was no active bleeding at the incision, the incision was repeatedly irrigated with normal saline and the wound was sutured layer by layer.

For patients undergoing hemiarthroplasty *via* PLA, they were placed in a conventional lateral position, and an arc-shaped incision of 10–14 cm was made with the greater trochanter as the center. The skin and subcutaneous fascia were incised, and then the gluteus maximus, gluteus medius, quadratus femoris, and flexion and internal rotation of the hip were bluntly separated. Next, the short external rotation muscle group was bluntly separated and severed at the greater trochanter of the femur. After cutting the joint capsule, the fractured end of the femoral neck was exposed, and the femoral neck was cut to leave about 1.0 cm of the femoral talus. The joint capsule was opened in different directions *via* a Hoffman retractor to clear the internal round ligament of the acetabulum. A grooving device and reamer device were utilized to open and ream the femoral medullary cavity, respectively. After installing the appropriately sized femoral prosthesis and femoral head, the hip joint was reset. When there was no dislocation of the hip joint, good stability, and no active bleeding at the incision, the short external rotator muscle group was sutured *in situ*, and the wound was closed layer by layer.

### Perioperative management

Routine preoperative fasting and drinking were prohibited, and low-molecular weight heparin calcium was discontinued 24 h before the operation. In addition, second-generation cephalosporin antibiotics and ammonia (20 mg/kg) were routinely used 30 min preoperatively. After the operation, the cocktail was used for a local injection on the wound surface. On the second postoperative day, patients were instructed to perform functional exercises. Anticoagulants were used to prevent deep vein thrombosis from 6 h after the operation to before discharge. Antibiotics were stopped on the second postoperative day. At 12–14 days postoperatively, depending on the wound healing, the sutures were removed.

### Clinical outcomes

The duration of the operation, length of the incision, blood loss, and postoperative hospitalization time were recorded. A visual analog scale (VAS) was utilized to evaluate the pain symptoms before and after surgery. The function of the affected hip joint was assessed using the Harris score. All patients were followed up for at least 6 months after surgery.

### Statistical analysis

Data were presented as mean ± standard deviation (SD) and analyzed using SPSS 19.0 software (SPSS, Chicago, IL, United States). The enumeration data were expressed as percentages and analyzed using a chi-square test. Perioperative data were compared using Student's *t* test. *P* < 0.05 was deemed to indicate a statistically significant difference.

## Results

### General characteristics

A total of 48 elderly patients with femoral neck fractures undergoing hemiarthroplasty were included in the present study. Of these, 24 cases were in the DSA group and 24 cases were in the PLA group. The baseline characteristics of the patients in the DSA and PLA groups are shown in Table 1. There were no obvious differences in age, gender, Garden type, American Society of Anesthesiologists (ASA) score, body mass index (BMI), or preoperative hematocrit between the two groups (*p* > 0.05).

**Table 1:** General characteristics.

	DSA (*n* = 24)	PLA (*n* = 24)	*P* value
Age (years)	84.54 ± 2.11	84.92 ± 2.15	0.544
Gender (*n*, %)			0.771
Male	13 (54.17)	14 (58.33)	
Female	11 (45.83)	10 (41.67)	
BMI (kg/m^2^)	20.73 ± 1.47	20.14 ± 1.02	0.112
Garden type (*n*, %)			0.745
Type III	6 (25.00)	7 (29.17)	
Type IV	18 (75.00)	17 (70.83)	
Preoperative hematocrit (%)	33.83 ± 3.63	34.00 ± 3.59	0.874
ASA score	1.96 ± 0.62	1.92 ± 0.65	0.822
Preoperative VAS score	6.00 ± 0.52	5.94 ± 0.51	0.688
Preoperative Harris score	41.50 ± 1.95	41.41 ± 1.99	0.875

DSA, direct superior approach; PLA, posterolateral approach; ASA, American Society of Anesthesiologists; BMI, body mass index; VAS, visual analog scale.

### Clinical outcomes

As presented in Table 2, the incision length in the DSA group was smaller than that in the PLA group (8.17 ± 0.92 vs. 12.67 ± 1.27 cm; *p* < 0.001). However, the duration of the operation in the DSA group was 76.58 ± 7.56 min and in the PLA group, it was 49.46 ± 7.68 min (*p* < 0.001). The blood loss in the DSA group was higher than that in the PLA group (197.08 ± 28.05 vs. 108.75 ± 26.92 ml; *p* < 0.001). After surgery, obvious differences were observed in postoperative hospitalization time, where the DSA group had a shorter hospitalization time compared to the PLA group (7.50 ± 1.25 vs. 12.46 ± 2.57 days; *p* < 0.001).

**Table 2:** Comparison of perioperative data between two groups.

	DSA (*n* = 24)	PLA (*n* = 24)	*p*-value
Incision length (cm)	8.17 ± 0.92	12.67 ± 1.27	<0.001
Duration of operation (min)	76.58 ± 7.56	49.46 ± 7.68	<0.001
Blood loss (mL)	197.08 ± 28.05	108.75 ± 26.92	<0.001
Postoperative hospitalization time (day)	7.50 ± 1.25	12.46 ± 2.57	<0.001

DSA, direct superior approach; PLA, posterolateral approach.

During the follow-up, we found that the VAS score at 1 month postoperatively in the DSA group was lower than that of the PLA group (1.78 ± 0.26 vs. 2.30 ± 0.25; *p* < 0.001). The Harris score in the DSA group was consistently higher than that in the PLA group 1 month postoperatively (84.58 ± 1.64 vs. 76.71 ± 3.64; *p* < 0.001). Moreover, there were no significant differences between the two groups in the Harris score 6 months postoperatively (86.42 ± 1.89 vs. 86.67 ± 1.81; *p* > 0.05) (Table 3).

**Table 3:** Comparison of main outcomes after operation.

	DSA (*n* = 24)	PLA (*n* = 24)	*p*-value
VAS score 1 month after surgery	1.78 ± 0.26	2.30 ± 0.25	<0.001
Harris score 1 month after surgery	84.58 ± 1.64	76.71 ± 3.64	<0.001
Harris score 6 month after surgery	86.42 ± 1.89	86.67 ± 1.81	0.642

DSA, direct superior approach; PLA, posterolateral approach; VAS, visual analog scale.

In addition, in the DSA group, one patient had an infection and was successfully treated by debridement and one patient died 1 year postoperatively due to pulmonary heart disease and other underlying diseases. In the PLA group, one patient had a periprosthetic fracture around the femoral stem due to a fall at home 8 months postoperatively and underwent surgery again and one patient died of lung infection 9 months postoperatively.

**Figure 1:**
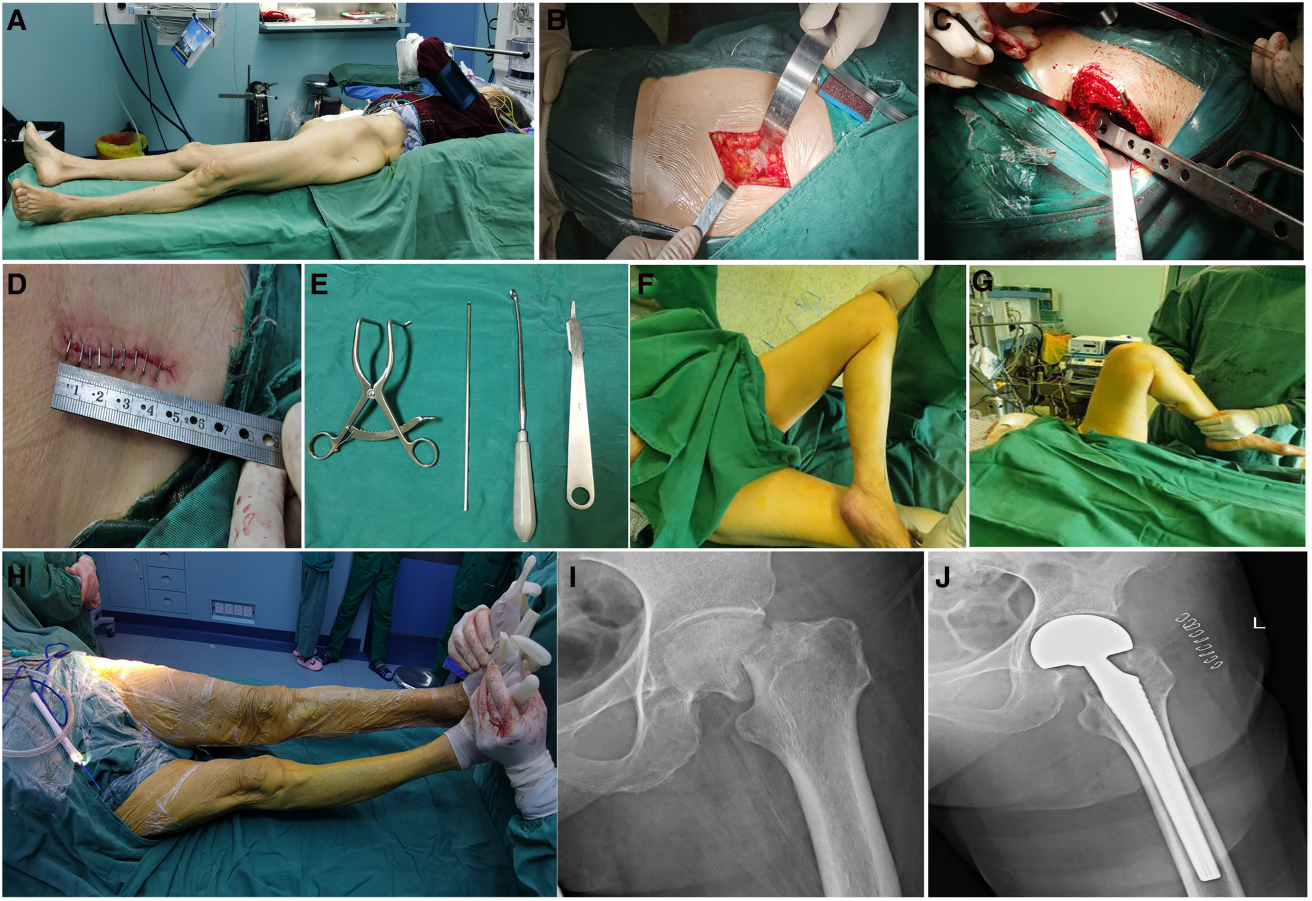
Representative case (87-year-old woman) of successful hemiarthroplasty using DSA. (**A**) Patient was placed in semi-recumbent position supine position on a surgical table. (**B**) A incision was made directly above the affected hip joint and superficial fascia was separated. (**C**) Femoral medullary cavity was grinded and reamed. (**D**) Special tools were required for the operation. (**E**) The length of the surgical incision was shown. (**F,G**) the movement of the hip joint after operation was inspected. (**H**) The lengths of lower limbs were compared afterward installing prosthesis. The x-ray images at preoperation (**I**) and postoperation (**J**).

The representative cases of successful hemiarthroplasty between the two groups are shown in Figure 1.

## Discussion

In the present study, we evaluated the treatment of displaced femoral neck fractures by using either DSA or PLA. The main finding was that hemiarthroplasty *via* DSA had a better clinical outcome in comparison with PLA for elderly patients with displaced femoral neck fractures, as demonstrated by the small surgical incision, mild early postoperative pain, shorter hospitalization time, and good early postoperative hip function.

It is worth mentioning that DSA enters through the gluteus maximus space, with the advantages of little damage and good rear stability (18). In addition, DSA surgery is similar to the posterolateral approach (19), and the short-term clinical efficacy is good (14). In the present study, we found that the patient positioning requirement was simple, without the need for a special operating bed and traction bed, and facilitated the management of anesthesia. However, DSA requires an experienced joint surgeon to perform the main operation due to the difficulty in exposing the lesser trochanter (20). In terms of operation time, a previous study has reported the longer operation time performing DSA compared to PLA in total hip arthroplasty (17). In accordance, our data displayed that the mean operation time was longer in the DSA group compared with the PLA group (76.58 ± 7.56 vs. 49.46 ± 7.68 min). We also observed that the blood loss in the DSA group was higher than in the PLA group (197.08 ± 28.05 vs. 108.75 ± 26.92 mL). The longer surgery time and higher blood loss may possibly be attributed to the learning curve of this new surgical technique.

Despite the longer mean surgery time, the postoperative hospitalization time was shorter in the DSA group compared with PLA group (7.50 ± 1.25 vs. 12.46 ± 2.57 days). In our cases, we observed that functional recovery was better in the DSA group. Evidence supporting the advantages of postoperative mobility in DSA compared to PLA is well established (17, 21) and may be explained by the minimally invasive technique with DSA, leading to reduced bleeding, faster healing time, rapid recovery, and a reduced risk of complications. In line with this, Tsiridis et al. (20) demonstrated that DSA can also be useful for hip dysplasia and in obese patients undergoing total hip arthroplasty. In our study, patients were aged older than 75 years, and we paid attention to the choice of surgical method. Importantly, the DSA approach can avoid the large deviation of length discrepancy in the lower limbs (22). The aforementioned findings indicate that hemiarthroplasty *via* DSA is suitable for elderly patients with displaced femoral neck fractures.

Pain relief is one of the main factors that increases patient satisfaction. In a previous study, Dorr et al. (23) stated that minimally invasive surgery results in better early pain relief. In addition, Renken et al. (24) also reported the obvious difference in VAS scores between another minimally invasive surgery (the direct anterior approach) and conventional treatment. Our study also displayed that the VAS score 1 month after surgery in the DSA group was a statistically significant improvement compared with that in the PLA group. This could be a result of the shorter length of incision and less tissue damage with DSA.

In terms of complications, a previous study reported that no sciatic nerve palsies, hip dislocations, or hip fractures were recorded in the total hip arthroplasty with the DSA approach (20). However, intraoperative calcar fractures and postoperative periprosthetic fractures occurred in the management of total hip arthroplasty *via* DSA (25). In agreement, no postoperative complications occurred in all patients with displaced femoral neck fractures undergoing hemiarthroplasty with PLA (26). Another study clarified that there was no obvious difference in the aspects of dislocation, mortality, and repeated operation after hemiarthroplasty between the PLA and lateral approach (27). Therefore, it is important to emphasize that the integrity of the external rotator muscles, such as the sinus piriformis, superior gemellus muscle, and inferior gemellus muscle, around the hip joint exert an important role in preventing dislocation after hip arthroplasty (28). In our study, no differences were observed when comparing postoperative complications or reoperation between the DSA and PLA groups. Among them, an 86-year-old patient in the PLA group had an improved quality of life and died 1 year postoperatively due to pulmonary heart disease. Another patient underwent a second debridement operation due to a superficial infection of the surgical wound. The main reasons for consideration were that the patient's long-term hypoalbuminemia and malnutrition led to delayed wound healing, and formation of a bursal cavity results in fluid secretion, as previously indicated (25). In the PLA group, one patient had a periprosthetic fracture around the femoral stem due to a fall at home 8 months postoperatively and underwent surgery again and one patient died of lung infection 9 months postoperatively. These findings suggested that underlying diseases and wound infection were the key to the recovery of hemiarthroplasty in elderly patients.

The limitations of this study are the small sample size and short follow-up time. Moreover, the data were retrospectively collected and some information, such as the presence of co-morbidities, were missed. Therefore, we could not analyze whether such confounders could have influenced our reported outcomes. Accordingly, further future prospective randomized trials with larger sample sizes are encouraged to further evaluate the efficacy of hemiarthroplasty with DSA for elderly patients with displaced femoral neck fractures.

DSA is less invasive and has better clinical outcomes, which can allow an early return to daily living activities in elderly patients with displaced femoral neck fractures undergoing hemiarthroplasty.

## Data Availability

The original contributions presented in the study are included in the article/Supplementary Material, further inquiries can be directed to the corresponding author.
